# Effects of ozone on zinc and cadmium accumulation in wheat – dose–response functions and relationship with protein, grain yield, and harvest index

**DOI:** 10.1002/ece3.423

**Published:** 2012-11-16

**Authors:** Håkan Pleijel

**Affiliations:** Department of Biological and Environmental Sciences, University of GothenburgP.O. Box 461, 405 30 Göteborg, Sweden

**Keywords:** Cadmium, dose–response function, open-top chamber, ozone, protein, wheat, zinc

## Abstract

Response functions for the effect of ozone on cadmium (Cd) (toxic to humans) and zinc (Zn) (essential nutrient for plants and humans) in wheat grain were derived for the first time. Data from four open-top chamber (OTC) experiments with field-grown wheat, performed in southwest Sweden, were used. Ozone exposure was expressed as the phytotoxic ozone dose above a threshold of 6 nmol/m^2^ per sec (POD_6_), and AOT40. Grain Zn concentration was significantly enhanced by ozone, while Zn yield was not affected. The positive ozone effect on grain Zn concentration was almost twice as large as the corresponding effect on grain protein concentration, most likely as a result of nitrogen availability being more limiting than Zn availability. Cd concentration was unaffected by ozone, but Cd yield was significantly negatively affected. For the variables studied, correlation was stronger with POD_6_ than AOT40, but in several cases, for example, for Zn concentration and Cd yield, there was practically no difference in the performance between the two exposure indices. From the literature, it is obvious that ozone has important adverse effects on wheat yield and certain quality traits. As shown in this study, there are also examples of ozone leading to improved quality, for example, in terms of enhanced Zn concentration of wheat grain. While OTC enclosure did not affect Zn accumulation in wheat grain, Cd accumulation was significantly positively affected, most likely through transpiration being enhanced by the OTC environment, promoting Cd uptake and transport through the plant.

## Introduction

One important aspect of food safety in wheat production is the cadmium (Cd) content of the grain. Cereals represent a key source of Cd for humans, which may have a number of important adverse health effects, for example, on kidney and bone (Satarug et al. 2010). Cd accumulation in wheat is affected by several factors, including genetic variation (Waters and Sankaran 2011), Cd concentration and availability in the soil (Guo et al. 2012), and factors influencing plant transpiration (Salt et al. 1995) because Cd is translocated from the root to the shoot in the transpiration stream of the xylem. Ozone (O_3_) can reduce the stomatal conductance of wheat (e.g., Pleijel et al. 2007), and thus limit transpiration of monocarpic plants like wheat by promoting leaf senescence (Grandjean and Fuhrer 1989; Ewert and Pleijel 1999). In addition, O_3_ negatively affects wheat grain yield, GY (Pleijel 2011). Reduced GY by O_3_ at a certain level of Cd uptake would enhance grain Cd concentration, while reduced transpiration as a consequence of elevated O_3_ is likely to reduce grain Cd concentration. Although the assessment of the balance of these counteracting factors is not simple, one could hypothesize that Cd concentration would remain largely unaffected by elevated O_3_, while Cd yield would be negatively affected as a result of lower GY and reduced transpiration.

The most commonly considered chemical quality aspect of wheat grain studied with respect to O_3_ exposure is grain protein, a nutritionally important trait, which is mostly calculated as crude protein from the nitrogen content of the grain. Several studies (e.g., Fangmeier et al. 1999; Piikki et al. 2008a) have shown that reduced GY of wheat is commonly associated with enhanced grain protein concentration (GPC). This represents in principle a positive influence on wheat grain quality. It can be explained as an effect of dilution/concentration of the nitrogen with an increase/decrease in biomass yield, a so-called growth dilution effect (Pleijel and Uddling 2012). However, over a large range of experiments, it has been shown that the unit area grain protein yield (GPY) in wheat is negatively influenced by O_3_ (Pleijel and Uddling 2012). Thus, the adverse effects of O_3_ on plant vitality leads to a reduction in nitrogen acquisition, but this effect is smaller than that on GY, leading to a net increase in GPC in response to O_3_.

Zinc (Zn) is an essential nutrient to the plant and is likewise an essential nutrient for humans. In some parts of the world, Zn deficiency is considered a major threat to food safety (Miraglia et al. 2009). Zn has been shown to be strongly correlated with GPC over a range of environmental conditions including different levels of O_3_ and CO_2_ (Pleijel and Danielsson 2009). Thus, it can be assumed that Zn would behave similarly to GPC in response to O_3_ exposure. However, although Zn limitation represents a serious problem in certain types of soil (Cakmak 2008), in most environments, nitrogen is more strongly limiting to the plant than Zn. Thus, the reduced N acquisition by O_3_-stressed plants (Pleijel and Uddling 2012) does not necessarily need to be associated with the same extent of reduction in Zn acquisition. A lesser degree of Zn than N limitation would suggest a larger positive effect of O_3_ on Zn concentration than on GPC and a smaller negative effect by O_3_ on Zn yield compared with GPY.

Potentially, rising levels of O_3_ may affect the accumulation of Cd and Zn in crops, thus influencing food safety (e.g., Miraglia et al. 2009). There are several studies of Cd uptake and effects on crops resulting from severe soil contamination with Cd, including interactive effects with O_3_ pollution (Di Cagno et al. 2001; Li et al. 2011; Guo et al. 2012). However, very few investigations have reported data reflecting the effect of O_3_ on crop Cd per se (but see Pleijel et al. 1991, 2006) and very little information seems to be available on the effect of O_3_ on Zn accumulation. No dose–response functions quantitatively linking crops Cd and Zn accumulation to O_3_ exposure seem to have been published.

The most widely used exposure indices for O_3_ effects on crops in Europe are AOT40 (the accumulated exposure over a threshold concentration of 40 nmol/mol; Fuhrer et al. 1997) and POD_6_ (the phytotoxic O_3_ dose above a threshold of 6 nmol/m^2^ per sec; Mills et al. 2011a). Although AOT40 only reflects the O_3_ concentrations surrounding the plants, POD_6_ is based on an estimation of the stomatal uptake of O_3_ as affected by different environmental variables such as temperature, vapor pressure deficit, and solar radiation. In direct comparison, POD_6_ mostly provided stronger relationships than AOT40 between experimentally observed effects and O_3_ exposure (Mills et al. 2011b), indicating a higher ecotoxicological relevance of the exposure index reflecting the physiologically controlled uptake of the pollutant by the plant.

**Figure 1:**
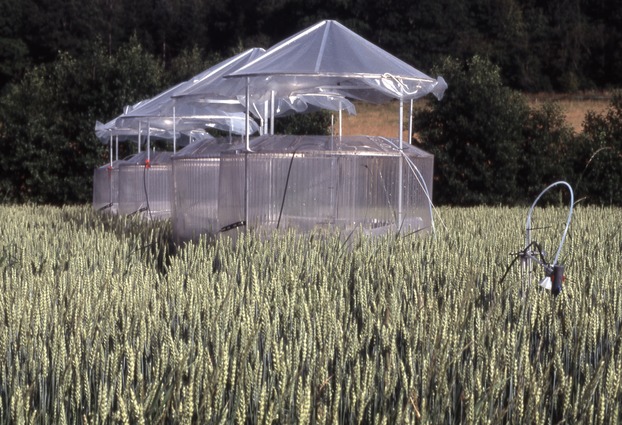
Picture showing the open-top chamber experimental system installed in a field of wheat, *Triticum aestivum* L. Photo by Håkan Pleijel.

The aim of the present investigation was to combine Cd and Zn data from open-top chamber (OTC) experiments with wheat (Fig. 1) performed in southwest Sweden to derive response functions for the effect of O_3_ on the concentration and yield of Zn and Cd in wheat grain. In addition, the effect of OTC enclosure on Zn and Cd concentration was evaluated, because OTCs can affect the microclimate of the plants (Piikki et al. 2008b). The hypotheses were as follows:

1. Wheat grain Zn concentration is increased by O_3_ exposure.
2. The effect of O_3_ on Zn concentration is larger than the effect on GPC and consequently Zn yield is less affected by O_3_ than GPY.
3. Wheat grain Cd yield is negatively affected by elevated O_3_.
4. Effects of O_3_ on wheat are more strongly related to POD_6_ than to AOT40.
5. OTC enclosure stimulates the accumulation of Cd in wheat grain as a result of enhanced transpiration.

## Materials and Methods

### Data

The data for this study were obtained from four OTC experiments performed at Östads säteri, southwest Sweden (57°540′N and 12°240′E). All experiments were performed with field-grown wheat crops (soil type: arenosol with loamy sand texture with the exception of the 1988 experiment which was a clay) following common agricultural practice. Cd data were available for all four experiments and Zn data for three experiments. The basics of the experiments have been described in the scientific literature (Pleijel et al. 1991, 1998, 2000, 2006). Only pure O_3_ treatments and data related to the modern wheat cultivars Drabant and Dragon were included. Thus, no interaction treatments with other environmental variables or data for the old landrace wheat variety “Lantvete” (Pleijel et al. 2006) were included.

An ambient air (AA) treatment was included in all experiments to monitor the effect of OTC enclosure. The 1988 experiment contained the following OTC treatments: charcoal-filtered air (CF), nonfiltered air (NF), and two levels of elevated O_3_ (NF+). The 1994 experiment had NF and three levels of NF+, the 1995 experiment NF and two NF+ treatments, whereas the 1999 experiment had CF and NF+. Thus, for the 1999 experiment, described in Pleijel et al. (2006), there was, unlike the other three experiments, no nonfiltered OTC treatment, but a filtered treatment with moderately reduced [O_3_]. The data presented in Table 2 to reflect the chamber enclosure effect for that data set were based on a comparison of the filtered treatment with the AA, whereas for the other experiments, AA were compared with NF. Air temperature was monitored at 0.1 m above the canopy in OTC and AA using Rotronic YA-100 sensors enclosed in radiation shields with forced ventilation.

The concentration of Zn was analyzed using induced coupled plasma atomic emission spectroscopy (ICP-AES). The Cd content was analyzed using induced coupled plasma mass spectrometry (ICP-MS), with the exception of the experiment described in Pleijel et al. (1991), where atomic absorption spectroscopy (graphite furnace) was used. Prior to the analysis, the samples were digested at a temperature of 550°C and then the elements were dissolved using HCl prior to dilution with HNO_3_. Nitrogen was determined using the Kjeldahl method. Protein concentration was obtained by multiplying the nitrogen concentration by 6.25.

In 1996, the soil content of Cd and Zn was analyzed in the AA and NF treatments (*n* = 6). The Zn content of the soil was 32 ± 6 mg/kg (mean ± standard deviation) in the AA treatment and 35 ± 3 mg/kg in the NF treatment. For Cd, the values were 0.14 ± 0.03 mg/kg in AA and 0.14 ± 0.02 mg/kg in NF. Soil pH varied between 6.0 and 6.4.

### Derivation of dose–response functions

Two exposure indices were included in the study. First, the phytotoxic ozone dose using a threshold of 6 nmol/m^2^ per sec (POD_6_) projected sunlit leaf area was used. It is described in detail in Mills et al. (2011a) and LRTAP Convention (2010). Second, the accumulated O_3_ exposure above a threshold of 40 nmol/mol (AOT40) was used for the same phenological period as defined by Mills et al. (2011a) for wheat. The AOT40 calculation is described in Fuhrer et al. (1997). The approach described by Fuhrer (1994) for calculating relative effects of O_3_ was used to combine the data from the different experiments on a common relative scale. The absolute level of each response variable at zero POD_6_ and AOT40, respectively, was estimated by linear regression for each experiment and response variable. The relative effect of O_3_ for all treatments of a certain experiment and variable was then calculated by dividing the value for each treatment with the estimated value for zero O_3_ exposure. In this way, linear dose–response functions for grain Zn concentration, Zn yield, Cd concentration, Cd yield, protein concentration, protein yield, GY, and harvest index (HI, calculated as the ratio between grain dry mass and total aboveground dry mass) using both POD_6_ and AOT40 were derived.

## Results

### Effects of O_3_ on Zn accumulation

**Figure 2:**
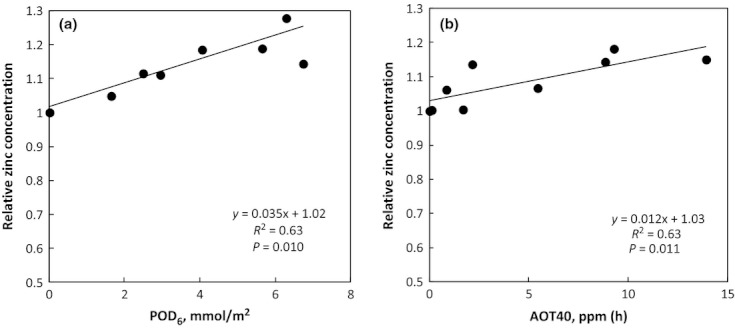
Linear relationships between the relative effect of ozone on Zn concentration and ozone exposure expressed as (a) POD6 and (b) AOT40.

**Figure 3:**
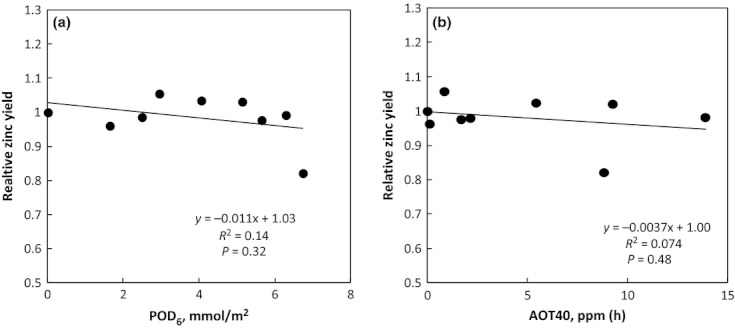
Linear relationships between the relative effect of ozone on Zn yield and ozone exposure expressed as (a) POD6 and (b) AOT40.

As evident from Figure 2a and b, there was a significant positive effect of O_3_ exposure on wheat grain Zn concentration. The *P*-values of regressions based on POD_6_ (Fig. 2a) and AOT40 (Fig. 2b) were almost identical, only slightly higher for POD_6_. The Zn yield was not affected by O_3_ exposure (Fig. 3a and b). The weak nonsignificant negative slopes for both the relationship with POD_6_ (Fig. 3a) and AOT40 (Fig. 3b) were driven by single points not representative for the overall pattern of the data indicating no response to O_3_ of this variable.

### Effects of O_3_ on Cd accumulation

**Figure 4:**
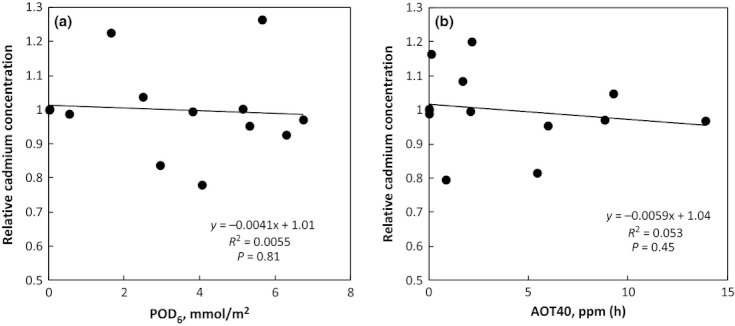
Linear relationships between the relative effect of ozone on Cd concentration and ozone exposure expressed as (a) POD6 and (b) AOT40.

**Figure 5:**
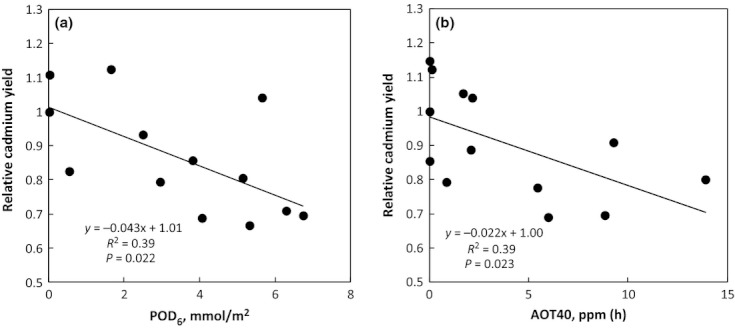
Linear relationships between the relative effect of ozone on Cd yield and ozone exposure expressed as (a) POD6 and (b) AOT40.

Figure 4a and b show the relationship between grain Cd concentration with POD_6_ and AOT40, respectively. These relationships are not significant and have large scatter, indicating a heterogeneous and nonsystematic response pattern of Cd concentration relative to O_3_ exposure. Cd yield, however, was significantly negatively influenced by O_3_ exposure (Fig. 5a and b). Both the relationship with POD_6_ (Fig. 5a) and that with AOT40 (Fig. 5b) were significant at the *P* < 0.05 level. The *P*-values of regressions based on POD_6_ (Fig. 2a) and AOT40 (Fig. 2b) were almost identical, but marginally higher for POD_6_. The slopes of these regressions were more strongly negative than for the corresponding relationships of GY with POD_6_ and AOT40 (Table 1): −0.043 versus −0.037 in the case of POD_6_ and −0.022 versus −0.015 for AOT40. Thus, there is an indication that Cd removal from agricultural land is more strongly negatively affected by O_3_ than GY, but the difference was not statistically significant.

**Table 1:** Characteristics of linear regression relationships of grain protein concentration (GPC), grain protein yield (GPY), grain yield (GY), and harvest index (HI) with ozone exposure expressed as the phytotoxic ozone dose above a threshold of 6 nmol/m^2^ per sec (POD_6_) or the accumulated exposure over a threshold of 40 nmol/mol (AOT40)

	POD_6_			AOT40		
		
	Linear equation	*R*^2^	*P*	Linear equation	*R*^2^	*P*
GPC	*y* = 0.021*x* + 1.00	0.88	<0.001	*y* = 0.008*x* + 1.02	0.56	0.015
GPY	*y* = −0.022*x* + 1.01	0.61	0.0016	*y* = −0.006*x* + 0.95	0.12	0.24
GY	*y* = −0.037*x* + 1.00	0.86	<0.001	*y* = −0.015*x* + 0.98	0.49	0.008
HI	*y* = −0.015*x* + 1.00	0.55	0.039	*y* = −0.014*x* + 1.09	0.30	0.054

### Effects of O_3_ on GPC, GPY, GY, and HI

In Table 1, response functions for GPC, GPY, GY, and HI, using both POD_6_ and AOT40 as O_3_ exposure index, are presented. While GPC was positively influenced by O_3_ exposure, GPY, GY, and HI were negatively affected. All response functions in Table 1 were statistically significant, except the GPY function with AOT40. All four effect variables proved to have stronger correlations with POD_6_ than AOT40, indicating that plant responses to O_3_ are more accurately explained by plant O_3_ uptake than by the O_3_ concentration in the air surrounding the plants.

### Comparison of O_3_ effects on Zn and Cd with effects on GPC

**Figure 6:**
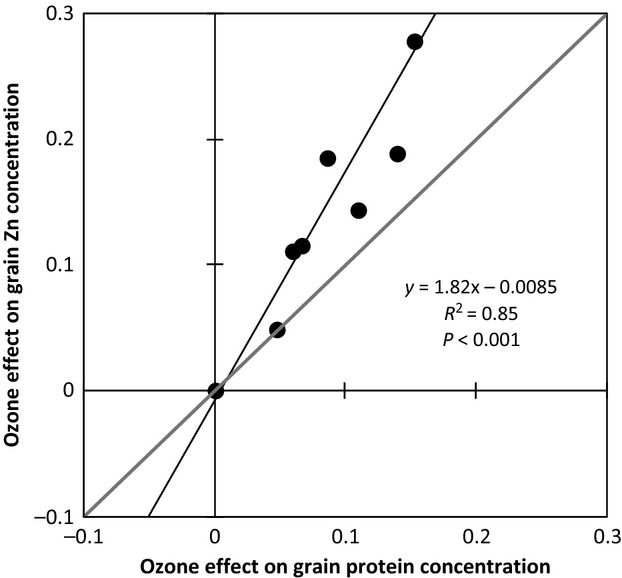
The effect of ozone (based on POD_6_) on Zn concentration in the different experimental treatments plotted versus the corresponding ozone effects on grain protein concentration (GPC). The bold gray line shows a hypothetical 1:1 relationship.

**Figure 7:**
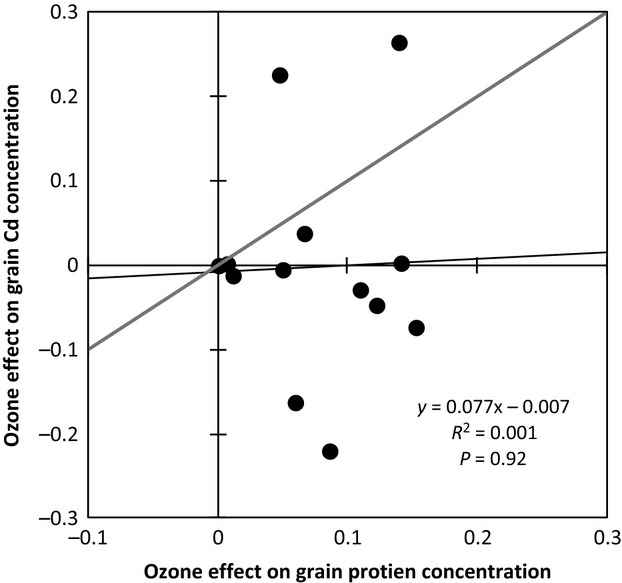
The effect of ozone (based on POD_6_) on Cd concentration in the different experimental treatments plotted versus the corresponding ozone effects on grain protein concentration (GPC). The bold gray line shows a hypothetical 1:1 relationship.

The slopes of the regressions between Zn concentration and O_3_ exposure (Fig. 2a and b) were steeper than the corresponding relationships for GPC (Table 1): 0.035 versus 0.021 in the case of POD_6_ and 0.012 versus 0.0076 for AOT40. For both indices, the difference was statistically significant (POD6: *P* = 0.005; AOT40: *P* = 0.014). Thus, it was shown that grain Zn is more strongly positively affected by O_3_ exposure than GPC. This was confirmed by plotting the effects by O_3_ (based on POD_6_) on Zn concentration versus the corresponding effects by O_3_ on GPC (Fig. 6) for all O_3_ treatments. A strong correlation (*R*^2^ = 0.85) was resulted, reflecting the close relationships between the O_3_ effect on grain Zn concentration and the effect on GPC over the range of O_3_ exposures used in the experiments. Furthermore, the slope of that regression was 1.82 with a very small intercept, indicating that the positive effect of O_3_ on Zn was almost twice the effect on GPC. The corresponding plot for Cd (Fig. 7) resulted in no clear relationship, indicating that the effects of O_3_ on GPC and Cd are essentially unrelated, the two processes being governed by different mechanisms.

### OTC effects on Zn and Cd

**Figure 8:**
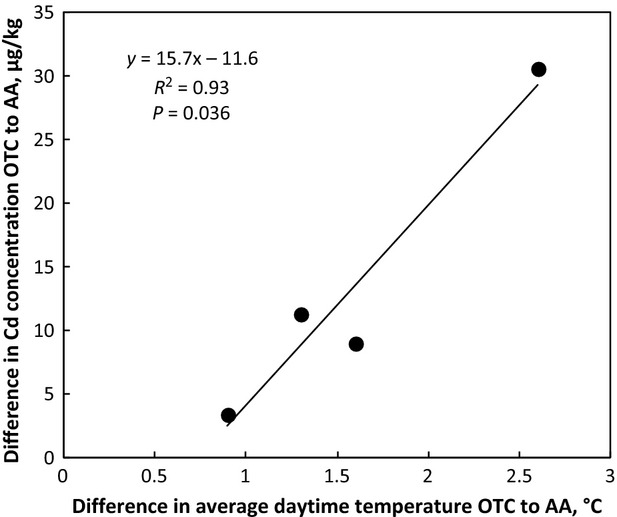
The difference in Cd concentration between the open-top chamber (OTC) control treatment and the ambient air (AA) treatment plotted versus the difference in daytime average temperature between the OTC and AA during the four experiments in which Cd was measured.

In Table 2, the effect of chamber enclosure on wheat grain Zn and Cd concentration is presented. In the case of Zn, there were only small nonsignificant (Student's *t*-test) differences between the OTC control treatment and AA. For Cd, on the other hand, there was a relatively large effect of OTC enclosure, the grain Cd concentration always being larger in the OTC compared with AA. This effect was statistically significant in three experiments out of four. In Figure 8, the difference in Cd concentration between OTC and AA is plotted against the corresponding difference in daytime average temperature. Although the relationship is based on a population of only four experiments, Figure 8 provides a strong indication that the OTC effect on Cd concentration is correlated with the OTC effect on temperature, the relationship being statistically significant.

**Table 2:** Grain concentrations of Zn (mg/kg) and Cd (μg/kg) expressed as average ± standard deviation (based on the OTC replicates), in the ambient air treatment (AA, no chamber enclosure) and the chamber control treatment of the experiment with the ozone concentration most similar to the AA (nonfiltered air in 1998, 1994, and 1995 and charcoal-filtered air in 1999). In addition, *P*-values from *t*-test of the difference between AA and the chamber control treatment are presented

	Zn			Cd		
		
	Chamber control	AA	*P*	Chamber control	AA	*P*
1988	–	–	–	56.2 ± 4.5^1^	47.2 ± 3.6^1^	0.0083
1994	33.3 ± 0.6	35.0 ± 1.2	0.46	59.3 ± 5.0	28.7 ± 5.5	0.0021
1995	23.6 ± 0.5	25.2 ± 0.2	0.24	36.6 ± 2.2	33.2 ± 4.3	0.23
1999	34.6 ± 5.4	31.3 ± 4.0	0.31	46.6 ± 4.7	35.3 ± 2.5	0.0028

1 From Pleijel et al. (1991).

## Discussion

In this study, a strong and significant positive effect of O_3_ on Zn concentration was observed based on O_3_ dose–response functions now derived for this essential nutrient. This result was in line with the first hypothesis and with earlier observations of enhanced concentrations of nitrogen (Fangmeier et al. 1999; Piikki et al. 2008a) and other minerals, for example, Ca, Mg, K, and P by Fuhrer et al. (1990); K and P by Vandermeiren et al. (1992); K and Ca by Feng et al. (2008); and Mg, P, and K by Pleijel et al. (2006), in response to O_3_ exposure. The slopes of the regressions for Zn concentration with O_3_ exposure were larger than for any other variable in this study, including GY, indicating the effect by O_3_ on Zn concentration to be comparatively strong.

In line with the general relationship between grain Zn and GPC found by Pleijel and Danielsson (2009) for a larger range of environmental conditions, there was a very strong correlation between effects of O_3_ on Zn concentration and GPC, both being essential nutrients to plants. However, the O_3_ effect on Zn concentration was almost twice as large as on GPC, most likely as a result of the fact that Zn availability is less limiting to plant uptake than N availability, and in line with the second hypothesis. Pleijel and Uddling (2012) showed that over a large range of experiments, although GPC was enhanced by O_3_, GPY was significantly negatively affected by O_3_. This reflects a reduction in acquisition of the strongly limiting nutrient N as a result of weakened plant vitality in response to O_3_ exposure. Zn, being less limiting in most agricultural situations, was less affected by O_3_. In fact, there was no effect by O_3_ on Zn yield in this study. This is in line with the assumption made by Loladze (2002), in an analysis of the effect of elevated CO_2_ on the concentration of plant nutrients, that the nutrient uptake remains constant over changes in GY. This assumption seems to be valid for the effect of nonlimiting Zn, but not for the strongly limiting N in the case of O_3_ effects based on the results from this study. It should be noted that in certain environments, especially high pH soils on calcareous ground (Cakmak 2008), Zn availability may be strongly limiting to plant uptake. Here, Zn may behave differently, the effect of O_3_ on Zn concentration may become smaller and the assumption of Loladze (2002) may not apply.

In this study, no significant effect of O_3_ on Cd concentration was observed. Cd yield was, however, negatively influenced and to a degree which was indicated to be larger than, for example, GY. This may be explained by the fact that transpiration is reduced by O_3_ exposure inducing and premature leaf senescence (Selldén and Pleijel 1995). The reduced Cd yield in response to O_3_ exposure was in agreement with the third hypothesis and means that the removal of Cd from agricultural land is reduced by O_3_ exposure of the crops.

For most response variables studied, the POD_6_ O_3_ exposure index (Mills et al. 2011a,b) performed better than AOT40 (Fuhrer et al. 1997). This exemplifies the stronger connection between O_3_ effect and stomatal O_3_ uptake than with the AOT40 index, which only reflects the [O_3_] outside the plants and, unlike POD_6_, does not include any physiological control of plant gas exchange (Mills et al. 2011b). This difference between POD_6_ and AOT40 was, however, essentially absent for certain biological effects, including some of those most in focus in this study, for example, Zn concentration and Cd yield. Thus, the fourth hypothesis was supported for GY, GPC, GPY, and HI, but not for Zn and Cd.

It has been suggested that the grain concentration of microelements like Zn may be related to HI. In a study of the trend for decreasing mineral density in wheat grain over a 160-year period, Fan et al. (2008) concluded that both increasing GY and HI were significant to explain the downward trend of grain mineral concentration. A higher HI, for example, as a result of plant breeding would lead to a more efficient redistribution of photosynthates than of minerals according to these authors, leading to lower levels of grain micronutrients. Conversely, reduced HI could be expected to lead to higher grain concentrations of micronutrients. In this study, HI showed a significant negative association with POD_6_ (Table 1), in line with earlier observations by Pleijel et al. (1995), while the Zn concentration was positively affected. However, the effects of O_3_ on Zn concentration did not correlate strongly with the corresponding effects on HI (*R*^2^ = 0.36, *P* = 0.089; data not shown). Thus, this study does not provide support for a strong link between O_3_ effects on Zn concentration and HI.

There is evidence that the uptake and accumulation of nonessential Cd is related to the transpiration stream through the plant (e.g., Salt et al. 1995). This was supported by this study in that OTC enclosure enhanced Cd concentration strongly (Table 2). This effect was proportional to the OTC effect on air temperature, supporting the fifth hypothesis. The OTC represents a warmer and thus in many cases dryer (higher vapor pressure deficit; Piikki et al. 2008b) than the AA. This promotes transpiration and can thus explain the observed effect of OTC enclosure on the Cd concentration. This is a potentially important observation in a climate change context, where higher temperatures may promote transpiration and thus enhance Cd accumulation in plants.

Many studies have shown that O_3_ has many adverse effects on crops and other plants. These include, in the case of wheat, lower GY (Pleijel 2011), reduced grain mass, and reduced volume weight (Piikki et al. 2008a). For grain protein, the situation is more complex as GPC has a clear positive response to O_3_ in most studies, including the present (Table 1) and several other (Fuhrer et al. 1990; Fangmeier et al. 1999; Piikki et al. 2008a), while GPY is negatively affected (Table 1; Pleijel and Uddling 2012). A reduced GPY may have serious consequences in areas where there is risk for protein deficiency among the population. Enhanced GPC on the other hand increases the price of wheat grain based on improved quality. In this study, Zn concentration was stimulated by O_3_ exposure more strongly than protein, whereas Cd concentration was not significantly affected. In conclusion, although the general picture of O_3_ effects on wheat is that most important traits studied (GY, GPY, grain mass, volume weight) are negatively affected, there exist also effects, which are beneficial from a quality/nutrition perspective (e.g., GPC and Zn concentration) and cases where O_3_ has no significant effect (e.g., Cd concentration). This is important in a food safety context, which is now receiving increasing attention with respect to global change (Miraglia et al. 2009).

It can be added that the absolute values of Cd concentration in this study (Table 2) did not exceed the maximum level of 200 μg/kg set by the EU regulation 420/2011 (EU 2011) for cereals.
